# School-level factors associated with teacher connectedness: a multilevel analysis of the structural and relational school determinants of young people’s health

**DOI:** 10.1093/pubmed/fdx089

**Published:** 2017-07-19

**Authors:** I García-Moya, F M Brooks, N H Spencer

**Affiliations:** 1 CRIPACC, School of Health and Social Work, University of Hertfordshire, College Lane Campus, Hatfield, Hertfordshire, UK; 2 Faculty of Health, University of Technology Sydney, PO Box 123 Broadway, Sydney, NSW, Australia; 3 Hertfordshire Business School, University of Hertfordshire, de Havilland Campus, Hatfield, Hertfordshire, UK

**Keywords:** educational settings, social determinants, young people

## Abstract

**Background:**

Conducting research on the antecedents of teacher connectedness (TC) is key to inform intervention and policy that can leverage the public health potential of teachers for young people’s well-being. As part of the EU-funded Teacher Connectedness Project, this study aims to examine the contribution of a variety of school-level factors (including type of school, school size, student–teacher ratio, students per class and teacher gender).

**Methods:**

Sample consisted of 5335 adolescents aged 11, 13 and 15 years that had participated in the HBSC study in England. Multilevel multinomial regression was used to examine the contributions of sociodemographic and school-level factors to TC.

**Results:**

TC was lower in older adolescents and those from less affluent families, but similar in boys and girls. Regarding school-level factors, it was not the size of the school but the ratio of students per teacher which was significantly associated to TC, with higher student–teacher ratio being significantly associated with lower odds of medium-to-high TC. Some differences between mixed and all-girls schools were also found.

**Conclusions:**

Health promotion strategies targeting student–teacher relationships need to consider how TC changes by age and SES and give attention to school-level factors, in particular the student–teacher ratio.

## Introduction

Schools are fundamental sites for young people not only from an educational point of view, but also from a public health perspective.^1^ The role of teachers in fostering students’ health and well-being is complementary to that of other health professionals in schools^2^ and teachers and the relationships they build with their students have been considered central for the effectiveness of public health initiatives at the school.^3^

An important domain in the current way school factors that act as determinants of health are conceptualized is the character of teacher–student relationships.^4^ Connectedness with both school and teachers have been associated with a variety of positive educational and health outcomes.^5–9^ The mechanisms by which school environment influences young people’s health are not currently completely understood but teacher connectedness (TC) seems to act both as a health asset^10^ and a protective factor, especially for the involvement in risk behaviours.^11^

The integrative theoretical approach by Bonell *et al.*^12^ suggests that school commitment, including the students’ attachment to the staff, shapes students’ affiliation with committed or non-committed peers, which will contribute to students’ cognitions and ultimately to their behavioural choices. Therefore, when relationships are built that foster commitment with school, young people are more likely to involve in pro-school activities and avoid risk behaviours.^12^ Qualitative research also points in this direction and underlines the key role of positive relationships with teachers in the process by which school environment affects young people’s health.^13^

School social ecology models assert that school connectedness results from the inter-relations between (i) structural and organizational factors and (ii) caring and supportive interpersonal relationships in the schools.^14^ Given that the school structural environment, such as school location and school composition are other important domains of school determinants of health,^4^ these inter-relations are worth exploring. Furthermore, relationships with teachers have shown potential as modifiable factors in interventions to decrease adolescent substance use^15,16^ and violence;^17^ therefore, it is important that efforts in this direction can take into consideration how structural and composition factors in the targeted schools may affect TC.

School characteristics, such as the size of the school, the demographics of their students or the neighbourhood the school is located in, socioeconomic status and school sector (government, independent and Catholic) have all been found to have an impact on school connectedness.^18–20^ School size has probably been the most extensively studied of these factors and a recent review suggests that developing and maintaining close relationships is easier for teachers in small schools, whereas higher disorganization and a more impersonal environment would make supportive student–teacher relationships less likely in large schools.^21^

However, one important challenge in the study of these school-level factors is that factors such as school size, sector, location, student composition and neighbourhood characteristics tend to be significantly interrelated,^22^ which makes it important to control for potentially confounding effects in this type of research. For example, the size of schools in rural areas tends to be smaller than in urban ones, but it has also been hypothesized that rurality may be associated with a more communitarian environment as opposed to the more bureaucratic one in urban schools; in other occasions an urban location has been linked to a higher prevalence of behavioural problems and violence.^23,24^

In addition, it seems advisable to further the study of school composition, which has tended to focus mostly on students.^25^ Teachers’ demographics especially gender may arguably be similarly important. For example, Bokhorst, Sumter and Westenberg^26^ hypothesized that the higher levels of teacher support found in girls in their study may have to do with a proportion of almost 80% female teachers in the Netherlands’ educational system and recommended the incorporation of teacher gender in future studies analysing teacher support. The relevance of examining the role of teachers’ gender is also supported by studies that describe significant differences in self-efficacy and teaching goals between female and male teachers,^27^ that may impact student–teacher connectedness. The relative number of teachers compared to students, which allows for calculating the student–teacher ratio, is also considered to be an important school-level indicator.^25,28^

Finally, an additional challenge in understanding the role of school-level factors for TC is that most studies have tended to subsume relationships with teachers within the broader concept of school connectedness (along with other aspects such as relationships with peers, liking school and school engagement).^18,19^ With the promotion of positive student–teacher relationships being increasingly seen as an effective strategy to promote students’ health at schools,^13,16^ the contribution of school factors to TC specifically warrants further attention.

As part of the EU-funded Teacher Connectedness Project ‘Well-being among European youth: The contribution of student–teacher relationships in the secondary-school population’, the overall aim of this study was to examine the potential contribution of a wide variety of school-level factors, including structural school characteristics and demographic composition, to TC. The Health Behaviour in School-aged Children (HBSC) study^29^ offers a nationally representative database based on both students’ and school staff’s data which includes information on both students’ perceptions of their relationships with teachers and information provided by the head, deputy head or subject head teacher on a number of school features that makes it especially suitable for this study’s aim.

## Methods

### Participants

Participants came from the representative sample in the 13/14 edition of the international WHO collaborative survey HBSC in England. A random sampling of all secondary schools in England stratified by region and type of school (state vs independent school) was used to obtain a nationally representative sample, which consisted of 5335 adolescents aged 11, 13 and 15 years from 48 schools (a total of 261 classes). From them, we selected the students from the 32 schools that had completed the school-level questionnaire (SLQ): 2927 adolescents (49.5% boys and 50.5% girls) aged 11–15 years. A more detailed description of the participants and their schools is provided as Supplementary Material.

### Measures

Variables were selected from two linked sources of data available in the HBSC study. Sociodemographic variables and TC measures were part of the self-completed questionnaires filled in by students, whereas information on school-level factors was collected by means of a separate SLQ completed by head, deputy head or subject head teacher in each of the participating schools. A description of the sociodemographic variables and school-level factors is presented in Table 1.

**Table 1:** Sociodemographic and school-level variables in the study

	Source	Categories	Rationale for coding and other observations
*Sociodemographic variables*			
Sex	HBSC students’ questionnaire	Boys Girls	–
Grade	HBSC students’ questionnaire	Year 7 Year 9 Year 11	–
Family affluence	HBSC students’ questionnaire:FAS-III^30^	Low (0–6) Medium (7–10) High (11–13)	Following recommendations on coding by Currie *et al.*,^31^ a sum score was obtained and categorized into three groups
*School-level factors*			
Type of school I	*HBSC SLQ*	Secondary school Middle school High school Grammar school Independent school	Secondary schools, middle schools and high schools are state-funded schools. Grammar schools are selective schools based on educational attainment. Independent schools are private schools
Type of school II	*HBSC SLQ*	All girls All boys Mixed	
Size of school	*HBSC SLQ*	<500 students Between 500 and 1000 students Between 1000 and 1500 students >1500 students	We used the information on number of students to create this categorical variable based on the current criteria for the description of school size in UK^32^
Student–teacher ratio	*HBSC SLQ*	NA	Quantitative variable derived from the information on total number of students and total number of teachers at the school
Female teachers	*HBSC SLQ*	NA	Percentage of female teachers out of the total number of teachers at the school
School location	*HBSC SLQ*	Village, hamlet or rural area (<3000 inhabitants) Small town (3000–15 000 inhabitants) Town (15 000–100 000 inhabitants) City (100 000 to 1 million inhabitants) Big city (>1 million inhabitants)	No recoding was done for this variable. Categories correspond with answer options provided in the SLQ
Neighbourhood problems in the school area	*HBSC SLQ*	NA	Respondents rated the degree of importance of 8 problems (tension based on racial, ethnic or religious differences, drug use, violence or vandalism…) in a Likert scale from 1 to 4. A sum score ranging from 8 to 32 was calculated
Migrant/minority students	*HBSC SLQ*	NA	Percentage of students in the school who ‘are ethnic or racial minority or have migration background’ and who ‘have a first language that is not English’
Student per class	*HBSC SLQ*	NA	Mean number of students per class

NA = Not applicable.

Regarding the dependent variable, TC was measured by means of the following three items on supportive teacher–student relationships, which are answered on a 5-point Likert scale from strongly disagree to strongly agree: ‘I feel that my teachers accept me as I am’, ‘I feel that my teachers care about me as a person’, and ‘I feel a lot of trust in my teachers’ Items in this scale were originally developed and validated within the international HBSC network^33^ and has been subjected to subsequent refinement and validation.^34^ Without explicit evidence to assume that the categories in the variable can be considered equally spaced on an underlying continuous scale, it can be inappropriate to treat it as continuous.^35^ Therefore, we used quartiles as a reference for recoding this ordinal variable into three groups to identify those with the lowest 25% of scores and those with the highest 25% of scores, leaving 50% in the middle category. Due to the grouped nature of the numbers underlying the scale, actual divisions were scores 1–3.33 as low (25.8%), 4.67–5 as high (30%), leaving 3.67–4.33 as middle (44.2%).

### Procedure

Data collection took place in the schools, students completed the questionnaires themselves and the confidentiality and anonymity of the data was ensured.^29^ Specifically, questionnaires were administered at schools either by teachers or members of the research team and students were asked to fill their questionnaires under exam type conditions; once completed, each student placed their questionnaire in an envelope and sealed it. Ethical approval was obtained from the University of Hertfordshire Ethics Committee for Health and Human Sciences (HSK/SK/UH/00007).

Regarding statistical analyses, multilevel multinomial regression modelling was carried out using MLwiN from within the R statistical package. Although TC was an ordinal variable, multilevel modelling of such dependent variables relies on estimation algorithms that sometimes lack stability. Initial modelling revealed that this was the case here. Treating the dependent variable as if it were simply multinomial solved such stability issues and an examination of the results did not show any inconsistencies introduced.

Size of the school, student–teacher ratio, female teachers and our two type of school indicators were initially included in the model based on our literature review. Additionally, stepwise selection (with the criterion for entry set at the 1% level of significance) was undertaken to investigate the possibility to include the additional school-level factors (location, neighbourhood problems, percentage of ethic or minority students, percentage of non-native speakers of English and mean number of students per class). Sex, grade and family affluence were also included in the model so that their potential confounding effects could be controlled for.

## Results

The obtained multilevel multinomial regression model of sociodemographic and school-level factors on TC is presented in Table 2. Grade, family affluence, student–teacher ratio, type of school II and school location were significantly associated with TC (*P* < 0.01). In contrast, TC was not significantly associated with sex, size of the school, percentage of female teachers and type of school I (secondary, middle, high, grammar or independent).

**Table 2:** Final multilevel multinomial regression model of sociodemographics and school-level factors on TC

	Coef.	*SE*	z	*P*-value
Sex (ref. category: boys)				
Girl-medium TC	−0.07995	0.09093	−0.88	0.3793
Girl-high TC	−0.13572	0.09819	−1.38	0.1669
Grade (ref. category: year 7)				
Year 9-medium TC	−0.95726	0.10382	−9.22	<0.001
Year 9-high TC	−1.90569	0.11348	−16.79	<0.001
Year 11-medium TC	−1.10295	0.10239	−10.77	<0.001
Year 11-high TC	−2.12378	0.11347	−18.72	<0.001
FAS (ref. category: low)				
Medium-medium TC	0.26107	0.11540	2.26	0.0237
Medium-high TC	0.34125	0.12611	2.71	<0.01
High-medium TC	0.18222	0.13190	1.38	0.1671
High-high TC	0.26871	0.14384	1.87	0.0617
Type of school I (ref. category: secondary)				
Middle-medium TC	0.78366	0.44035	1.78	0.0751
Middle-high TC	−0.03311	0.47085	−0.07	0.9439
High school-medium TC	−0.19254	0.28942	−0.67	0.5059
High school-high TC	−0.18303	0.30482	−0.60	0.5482
Grammar-medium TC	0.16212	0.37244	0.44	0.6634
Grammar-high TC	0.75193	0.40832	1.84	0.0655
Independent-medium TC	0.67561	0.27412	2.46	0.0137
Independent-high TC	0.67630	0.28953	2.34	0.0195
Type of school II (ref. category: all girls)				
All boys-medium TC	0.18491	0.41318	0.45	0.6545
All boys-high TC	0.55727	0.44584	1.25	0.2113
Mixed-medium TC	0.91149	0.26451	3.45	<0.001
Mixed-high TC	1.08858	0.28801	3.78	<0.001
Location (ref.category: village, hamlet or rural area)				
Small town-medium TC	0.69846	0.33268	2.10	0.0358
Small town-high TC	0.53970	0.35742	1.51	0.1310
Town-medium TC	0.92756	0.32621	2.84	<0.01
Town-high TC	0.67863	0.35106	1.93	0.0532
City-medium TC	0.42581	0.26189	1.63	0.1040
City-high TC	0.32142	0.27549	1.17	0.2433
Large city-medium TC	0.40917	0.35696	1.15	0.2517
Large city-high TC	0.21385	0.38169	0.56	0.5753
Size of school (ref. category: <500 students)				
Between 500 and 1000-medium TC	0.25624	0.27758	0.92	0.3559
Between 500 and 1000-high TC	0.58227	0.29366	1.98	0.0474
Between 1000 and 1500-medium TC	0.33053	0.29671	1.11	0.2653
Between 1000 and 1500-high TC	0.55307	0.31334	1.77	0.0776
>1500-medium TC	−0.15455	0.27066	−0.57	0.5680
>1500-high TC	−0.19174	0.28412	−0.67	0.4998
Student–teacher ratio				
Student–teacher ratio-medium TC	−0.14008	0.03359	−4.17	<0.001
Student–teacher ratio-high TC	−0.17514	0.03663	−4.78	<0.001
Female teacher				
Female teacher-medium TC	−0.00209	0.00921	−0.23	0.8209
Female teacher-high TC	0.01234	0.01031	1.20	0.2316

The addition of interactions between significant main effects to the model was investigated but none could be added reliably.

Descriptives and ORs and their 95% CIs for each significant variable in all relevant comparisons are presented in Table 3 and Table 4, respectively. It must be noted that we decided to restrict the description of significant results to those comparisons where the *P*-value was <0.001 because of the large number of comparisons that were carried out. At this point, the variable location showed no strong overall patterns (*P* < 0.001) and, therefore, further analysis of this variable is not presented.

**Table 3:** Percentages of low, medium and high TC by grade, FAS and type of school (all-girls, all-boys, mixed) and student–teacher ratio means by TC

TC	Low	Medium	High
Grade: Year 7	10.8%	41.3%	47.9%
Grade: Year 9	33.6%	44.8%	21.6%
Grade: Year 11	36.1%	44.3%	19.6%
Low FAS	30.5%	41.5%	28.0%
Medium FAS	25.3%	44.2%	30.5%
High FAS	24.3%	44.4%	31.3%
Type of school: All-girls	32.7%	37.4%	29.9%
Type of school: All-boys	15.1%	46.8%	38.1%
Type of school: Mixed	26.1%	43.6%	30.3%
Student–teacher ratio	13.11	12.68	12.51

**Table 4:** Odds ratios with 95% confidence intervals for grade, FAS, type of school (all-girls, all-boys, mixed) and student–teacher ratio

	Medium vs Low TC		High vs Low TC		High vs Medium TC	
	OR (95% CI)	*P*	OR (95% CI)	*P*	OR (95% CI)	*P*
Grade						
Year 9 vs Year 7	0.38 (0.31,0.47)	<0.001	0.15 (0.12,0.19)	<0.001	0.39 (0.26,0.57)	<0.001
Year 11 vs Year 7	0.33 (0.27,0.41)	<0.001	0.12 (0.10,0.15)	<0.001	0.36 (0.25,0.53)	<0.001
Year 11 vs Year 9	1.16 (0.95,1.41)	0.148	1.24 (0.98,1.58)	0.075	1.08 (0.74,1.57)	0.708
FAS						
Medium vs Low	1.30 (1.04,1.63)	0.024	1.41 (1.10,1.80)	<0.001	1.08 (0.71,1.66)	0.714
High vs Low	1.20 (0.93,1.55)	0.167	1.31 (0.99,1.73)	0.062	1.09 (0.67,1.78)	0.730
High vs Medium	1.08 (0.90,1.30)	0.407	1.08 (0.88,1.31)	0.477	1.08 (0.74,1.57)	0.708
Type of school II						
All boys vs All girls	1.20 (0.54,2.70)	0.654	1.75 (0.73,4.18)	0.211	1.45 (0.31,6.76)	0.635
Mixed vs All girls	2.49 (1.48,4.18)	<0.001	2.97 (1.69,5.22)	<0.001	1.19 (0.45,3.18)	0.723
All boys vs Mixed	0.48 (0.23,1.02)	0.058	0.59 (0.26,1.31)	0.194	1.22 (0.29,5.08)	0.789
Student–teacher ratio						
1-unit increase	0.87 (0.81,0.93)	<0.001	0.84 (0.78,0.90)	<0.001	0.97 (0.85,1.09)	0.582
5-unit increase	0.50 (0.36,0.69)	<0.001	0.42 (0.29,0.60)	<0.001	0.84 (0.45,1.57)	0.582

Regarding sociodemographic variables, adolescents in years 9 and 11 had lower odds of reporting medium (compared to low) and high (compared to medium or low) levels of TC than those in year 7 (see Table 4); those in year 7 were more represented in the medium and high TC categories and underrepresented in the low level compared to older adolescents (see Table 3). In addition, adolescents with a low FAS seemed to some extent overrepresented in the low TC category (see Table 3). ORs suggested that, compared to adolescents with low FAS, those with medium FAS had significant greater odds to report high than low TC. In contrast, no significant differences in the odds of reporting high (compared to medium and low) or medium (compared to low) TC were found between adolescents with high and medium FAS.

As for significant school-level factors, the student–teacher ratio mean tended to be higher in the schools of adolescents reporting low TC (see Table 3). ORs indicated that higher student–teacher ratio was significantly associated with lower odds of high or medium (compared to low) TC. As apparent when looking at ORs for 1-unit and 5-unit increases, as student–teacher ratio increased the likelihood of medium to high TC decreased. Finally, regarding type of school, adolescents in mixed schools were more likely to show medium or high TC compared with those in all-girls schools. The rest of comparisons in this variable did not reach statistical significance.

## Discussion

### Main finding of this study

TC was lower in adolescents from higher grades (school years) and less affluent families, however, strongly similar among both boys and girls. Regarding school-level differences, we found that low TC was more likely as student–teacher ratio increased and less likely in mixed schools compared to all-girls schools. These school-level factors were found significant once students’ sex, age and SES had been controlled for.

In contrast, TC did not significantly vary depending on type of school (secondary, middle, high school, grammar and independent), school location, size of the school or mean number of students per class, percentage of migrant/minority students, percentage of female teachers and neighbourhood problems in the school area.

### What is already known on this topic

Previous studies have indicated that school connectedness and TC have a significant impact in young people’s health^7,10^ and that it tends to be lower in older adolescents.^10,26^ It also seems that low SES is associated with lower teachers’ expectations^36^ as well as with students’ lower educational outcomes and higher behavioural problems, with all these aspects influencing one another probably resulting in the lesser closeness in student-teacher relationships.^37^ Research has also suggested that school characteristics can make a significant difference, with a smaller school size having been one of the most consistently reported factors favouring young people’s school connectedness.^18,19,21^

On a different but related matter, recent reviews and seminal papers have called for greater conceptual clarity around school connectedness^38,39^ and the need to break down such a broad concept into the aspects that are important,^40,41^ messages which to date do not seem to have been sufficiently incorporated into research on the role of school characteristics.

### What this study adds

This study took on board the aforementioned developments in the area and looked to the role of school characteristics in TC specifically, as a distinct and central element of school experience on its own.

Our findings suggest that it is not the absolute size of the school but the ratio of students per teacher which is significantly associated with TC, which significantly adds to previous research. Student–teacher ratio can affect TC via both direct and indirect mechanisms. Individualized interactions with students^42^ and the extent to which teachers notice students and know them at a personal level^43^ are important aspects of TC, and student–teacher ratio is significantly linked with the opportunities for these types of interactions at a school. Student–teacher ratio may also have indirect effects in the quality of student–teacher relationships because high ratios can result in teachers’ overload and stress.^44^ Small schools usually have lower ratios, but what our findings suggest is that it is an appropriate balance between the numbers in the student and teacher bodies that matters, i.e. that TC can also be fostered in large schools as far as they are sufficiently staffed.

In addition, in our sample, mixed schools were more likely to show high TC than all-girls schools, after controlling for potentially confounding effects of students’ demographics. Our findings in this respect must be cautiously interpreted because only four all-girls schools had taken part in the study; however, this is an interesting aspect to which research on single-sex vs mixed schools have paid little attention.^45^ The fact that school gender composition is related to many other variables makes it challenging to identify clear effects, but as mentioned by Smithers and Robinson ‘single-sex schools and co-educational schools can look and feel very different’,^46^ which makes one wonder whether that might have some impact on teacher–student interactions, an aspect which deserves further examination in future research.

Finally, our findings have important implications for public health. TC and most of the school-level factors examined in the present study are modifiable determinants of health, which means that they can be influenced by policy. For example, findings pointing to school size as a key variable led to initiatives in the US to divide large comprehensive schools into multiple smaller schools, so that structures facilitated personalization and strong relationships.^47^ Our results on the importance of student–teacher ratio over school size suggests that a better measure for the promotion of TC may be making sure that no school, large or small, is understaffed. In the UK context, where size of schools is highly influenced by parental demand and there is a relative freedom of the schools to determine their size,^48^ attention to student–teacher ratio is particularly important. Accordingly, the design and investment in effective recruitment and retaining policies should be a priority in educational policy. On the other hand, school location or type of schools (comparing state-funded and private schools as well as selective and non-selective schools), to name some examples, were not significant and therefore initiatives focusing on them are not likely to result in increases in TC. Regarding the role of co-educational vs single-sex schools our results are not conclusive and further research is recommended.

More broadly, the attention to TC as an important determinant of health calls for greater collaboration between the public health and educational sectors. With the potential of student–teacher interactions as protective for risk behaviours^15–17^ and the emerging concerns about mental health among youth,^49,50^ the role of education systems as public health institutions and as sites for public health interventions become vitally important. It is therefore fundamental that public health adopts a more nuanced understanding of the elements within schools that operate as determinants of health and the links between them. In order to overcome the traditional disconnection between health and educational goals at schools,^51^ work should be done via education policy and in collaboration between public health professionals and school leaders to make sure our schools’ system, culture and ethos contribute to young people’s health and well-being.

### Limitations of this study

A main limitation of this study is its cross-sectional design, which does not allow for drawing conclusions about the direction of the associations found.

Secondly, although the HBSC database was particularly useful for the aim of this study and the multilevel analyses conducted respected the nested nature of student- and school-level data, sampling focused on the representativeness of the students’ sample, and therefore did not ensure a balanced representation of certain types of schools, such as the abovementioned all-girls schools, which limits the generalization of that finding.

Finally, mixed method research may be useful to test some of the interpretations of our findings and get a deeper view of the perceptions of teachers and students on the ways and mechanisms through which school factors can impact student–teacher connectedness.

## Supplementary Material

Supplementary Data
