# Supplementary material for: School-level factors associated with teacher connectedness: a multilevel analysis of the structural and relational school determinants of young people’s health
Source: J Public Health (Oxf). 2017 Jul 19;40(2):366–74. doi: 10.1093/pubmed/fdx089 (PMC6051442; doi:10.1093/pubmed/fdx089)
Supplement: Supplementary Data [file supplementarymaterial.docx]

**Supplementary material.** *Sociodemographic and school characteristics in the study sample*

| **Sociodemographic characteristics** | **N** | **Descriptives** |
| --- | --- | --- |
| Boys | 1450 | 49.5% |
| Girls | 1477 | 50.5% |
| 11 years | 1120 | 38.3% |
| 13 years | 872 | 29.8% |
| 15 years | 935 | 31.9% |
| Low family affluence | 429 | 16.8% |
| Medium family affluence | 1355 | 53.1% |
| High family affluence | 768 | 30.1% |
| **School characteristics** | **N** | **Descriptives** |
| Secondary school | 1943 | 66.4% |
| Middle school | 106 | 3.6% |
| High school | 107 | 3.7% |
| Grammar school | 90 | 3.1% |
| Independent school | 681 | 23.3% |
| All-girls school | 321 | 11.0% |
| All-boys school | 299 | 10.2% |
| Mixed school | 2307 | 78.8% |
| Located in a village, hamlet or rural area (< 3,000 inhabitants) | 278 | 9.5% |
| Located in a small town (3,000-15,000 inhabitants) | 505 | 17.3% |
| Located in a town (15,000-100,000 inhabitants) | 967 | 33.0% |
| Located in a city (100,000 to 1million inhabitants) | 603 | 20.6% |
| Located in a big city (> 1 million inhabitants) | 574 | 19.6% |
| Percentage of migrant/minority students | 2927 | *M* = 27.90 |
| Less than 500 students | 365 | 12.5% |
| Between 500 and 1000 students | 1162 | 39.7% |
| Between 1000 and 1500 students | 767 | 26.2% |
| More than 1500 students | 633 | 21.6% |
| Student-teacher ratio | 2927 | *M* = 12.74 |
| Students per class | 2570 | *M* = 23.58 |
| Percentage of female teachers | 2927 | *M* = 60.93 |
| Neighbourhood problems in the school area (range: 8 to 32) | 2346 | *M* = 15.39 |
